# The tobacco carcinogen NNK drives accumulation of DNMT1 at the GR promoter thereby reducing GR expression in untransformed lung fibroblasts

**DOI:** 10.1038/s41598-018-23309-2

**Published:** 2018-03-20

**Authors:** Kerryn M. Taylor, Roxanne Wheeler, Nimisha Singh, Dalene Vosloo, David W. Ray, Paula Sommer

**Affiliations:** 1 School of Life Sciences, University of KwaZulu-Natal, Durban, South Africa; 2 Division of Endocrinology, Diabetes and Gastroenterology, School of Medical Sciences, Faculty of Biology, Medicine and Health, University of Manchester, Manchester, United Kingdom

## Abstract

Small cell lung cancer (SCLC) is a highly aggressive, predominantly cigarette smoke-induced tumour with poor prognosis. The glucocorticoid receptor (GR), a SCLC tumour suppressor gene, is typically reduced in SCLC. We now show that SCLC cells express high levels of DNA methyltransferase 1 (DNMT1) which accumulates at the GR promoter. DNMT1 expression is further increased by exposure to the tobacco carcinogen NNK. In the untransformed human lung fibroblast cell line, MRC-5, short term NNK treatment decreases GRα mRNA and protein expression due to accumulation of DNMT1 at the GR promoter. Long term NNK treatment results in persistently augmented DNMT1 levels with lowered GR levels. Long term exposure to NNK slows cell proliferation and induces DNA damage, while the GR antagonist RU486 stimulates proliferation and protects against DNA damage. Although both NNK and RU486 treatment increases methylation at the GR promoter, neither are sufficient to prevent senescence in this context. NNK exposure results in accumulation of DNMT1 at the GR promoter in untransformed lung cells mimicking SCLC cells, directly linking tobacco smoke exposure to silencing of the GR, an important step in SCLC carcinogenesis.

## Introduction

Small cell lung cancer (SCLC) is an aggressive neuroendocrine cancer that is cigarette smoke-induced in >95% of cases^1^. SCLC tumours secrete the adrenocorticotrophin hormone (ACTH) precursor, proopiomelanocortin^2^, and other ACTH-related peptides. However in contrast to normal production by the pituitary, this ectopic ACTH secretion is unresponsive to the negative feedback of glucocorticoids (Gcs)^3,4^. This resistance to Gc inhibition can be attributed to deficient expression of the glucocorticoid receptor (GR) in the SCLC cells^4,5^.

The GR (NR3C1) is an ubiquitously expressed transcription factor encoded by a single copy gene on chromosome 5^6,7^. Its promoter region comprises nine alternative promoters, the proximal seven of which (1B-1F, 1H and 1J) are located in a ~3.1 kb CpG island^8,9^. We have previously shown that GR expression in SCLC cell lines is reduced due to aberrant methylation of the proximal promoters, 1D and 1E, with extensive methylation of promoters 1F and 1C^10,11^. Treatment with a demethylating agent leads to re-expression of GRα (the major functional translational isoform) protein^10^, mediated by promoters 1B, 1C and 1J, but predominantly 1F^11^. Importantly, the GR is an authentic tumour suppressor gene (TSG)^12^ and restoration of endogenous GR expression drives the SCLC cells to apoptosis^11^. Loss of GR expression thus confers a survival advantage to the cells.

Epigenetic silencing of TSGs is causally linked with tumourigenesis^13,14^. The tobacco-specific carcinogen, NNK (4-(methylnitrosamino)-1-(3-pyridyl)-1-butanone; also known as nicotine-derived nitrosamine ketone), has been directly linked to the hypermethylation of numerous TSGs in lung cancers whereby exposure to NNK leads to nuclear accumulation of DNA methyltransferase 1 (DNMT1) at gene promoters, resulting in promoter hypermethylation. DNMT1 is typically over-expressed in lung and liver cancer patients that smoke, with high expression levels correlating with poor prognosis^15^. Over-expression of DNMT1 in normal cells can cause aberrant *de novo* methylation of promoter-CpG islands and malignant transformation^16,17^. Therefore NNK-induced DNMT1 accumulation associated with epigenetic silencing of TSGs may lead to carcinogenesis. This mechanism provides another significant link between tobacco smoking and lung cancer^15^.

The aim of this study was to explore the mechanism of epigenetic modification in SCLC and non-cancerous lung cells, by investigating whether exposure to NNK induces up-regulation of expression and accumulation of DNMT1 at the GR promoter, resulting in silencing of GR expression.

## Results

### Endogenous DNMT1 accumulation at GR promoter 1F and elevated DNMT1 expression following NNK exposure in SCLC cells

**Figure 1:**
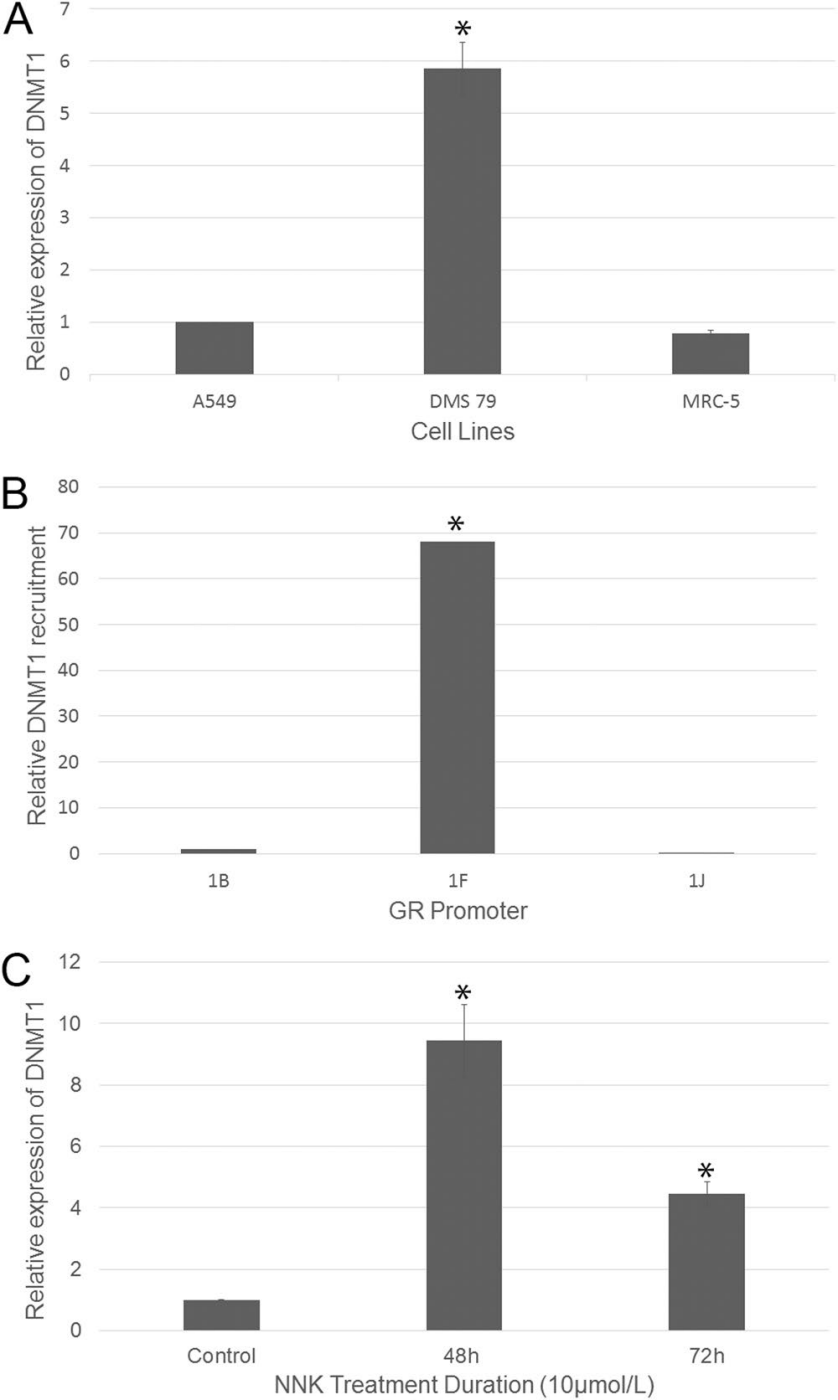
SCLCs express high levels of DNMT1 which accumulate at the GR promoter 1F. DNMT1 expression is increased further in the presence of the tobacco carcinogen, NNK. (**A**) Relative expression of DNMT1 in A549, DMS79 and MRC-5 cell lines (n = 3, p < 0.05 compared to other cell lines). (**B**) ChIP analysis shows accumulation of DNMT1 at the GR promoter 1F in DMS79 cells (n = 3, p < 0.05 compared to promoter 1B and 1J). (**C**) Relative expression of DNMT1 in DMS79 cells treated with NNK for 48 and 72 h (n = 3, p < 0.05 compared to the untreated DMS79 control). qPCR data were normalized to two reference genes, GAPDH and β-actin, and the geometric mean ± standard error is shown.

The SCLC cell line, DMS79, shows significantly higher expression levels of DNMT1, the enzyme responsible for methylation, than the non-SCLC (NSCLC) cell line, A549, and untransformed human lung fibroblast line, MRC-5 (Fig. 1A). Previously, we showed that the ubiquitously expressed GR is silenced by methylation in SCLC cell lines and xenografts and that silencing may play a role in carcinogenesis^4,10,11,18^. We now show in DMS79 cells that DNMT1 is recruited to the GR promoter 1F (Fig. 1B). Since smoking is the predominant cause of lung cancers, we investigated whether further exposure of SCLC cells to the tobacco carcinogen, NNK, would affect DNMT1 levels, and indeed it did, especially after 48 h (Fig. 1C).

### The effects of short-term NNK treatment on GRα and DNMT1 expression in MRC-5 cells

**Figure 2:**
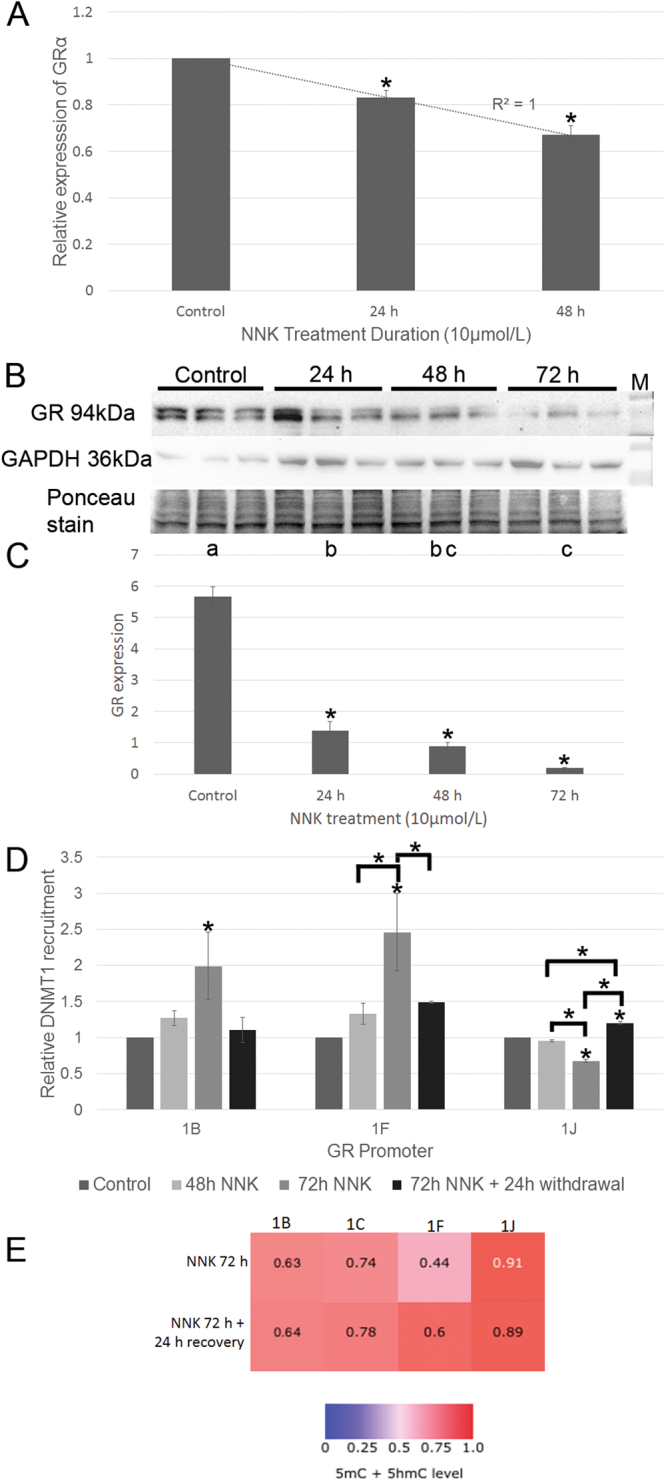
Short term NNK treatment of the untransformed human lung cell line, MRC-5, results in a decrease in GRα mRNA and protein expression due to NNK-induced DNMT1 accumulation at the GR promoter 1B and 1 F. (**A**) Relative expression of GRα mRNA in MRC-5 cells after 10 µmol/L NNK exposure for 24 and 48 h measured by qPCR (n = 3, p < 0.05 compared to the untreated control). (**B**) Western blot analysis of GRα protein expression after 24, 48 and 72 h NNK treatment. Each sample is repeated in triplicate. The top panel is a continuous, uncropped image of GR exposure, the middle panel shows GAPDH while the bottom panel is Ponceau S staining of the GR region. (**C**) Densitometric analysis of GR expression relative to GAPDH (*p < 0.05 compared to the control, different letters represent statistical differences between treatments). (**D**) ChIP analysis shows accumulation of DNMT1 at the GR promoters 1B and 1F (n = 3, p < 0.05 compared to each promoter control, other statistical differences are indicated). (**E**) Heat map of mean GR promoter methylation levels in MRC-5 cells treated with 72 h NNK, or 72 h NNK plus a 24 h recovery in fresh media. Methylation depicted includes methylation (5 mC) and hydroxymethylation (5 hmC), the principal methylation-associated epigenetic modifications.

Previous studies have shown that NNK silences TSGs by accumulation of DNMT1 at their promoters^15^. The GR has recently been described as a novel TSG^12^. We thus investigated whether NNK exposure would affect GR expression in MRC-5 cells, a non-cancerous, karyotypically normal human lung fibroblast cell line that typically senesces after 42–46 doublings^19^. Our data show that NNK treatment significantly reduces GRα expression at both mRNA and protein level (Fig. 2A, B and C). Our data show that there is significant recruitment of DNMT1 to GR promoters 1B and 1F after 72 h NNK exposure (Fig. 2D), and high levels of methylation at promoters 1B, 1 C, 1F and 1J relative to the control (Fig. 2E) (methylation was calculated with reference to the MRC-5 72 h vehicle-treated cells). Since the methylation status of this sample is indeterminate (without a reference itself) it is not included in the output. DNMT1 recruitment and demethylation following 24 h NNK withdrawal appears to be delayed (Fig. 2D and E). Analysis of the TCGA database (https://cancergenome.nih.gov) revealed no excess of mutations, of copy number variations, in the DNMT1 or GR (NR3C1) gene compared to the index NCI60 cell line panel, further support that the changes seen are epigenetic.

### Chronic NNK and RU486 exposure attenuates GRα and elevates DNMT1 expression while RU486 has a protective function in MRC-5 cells

**Figure 3:**
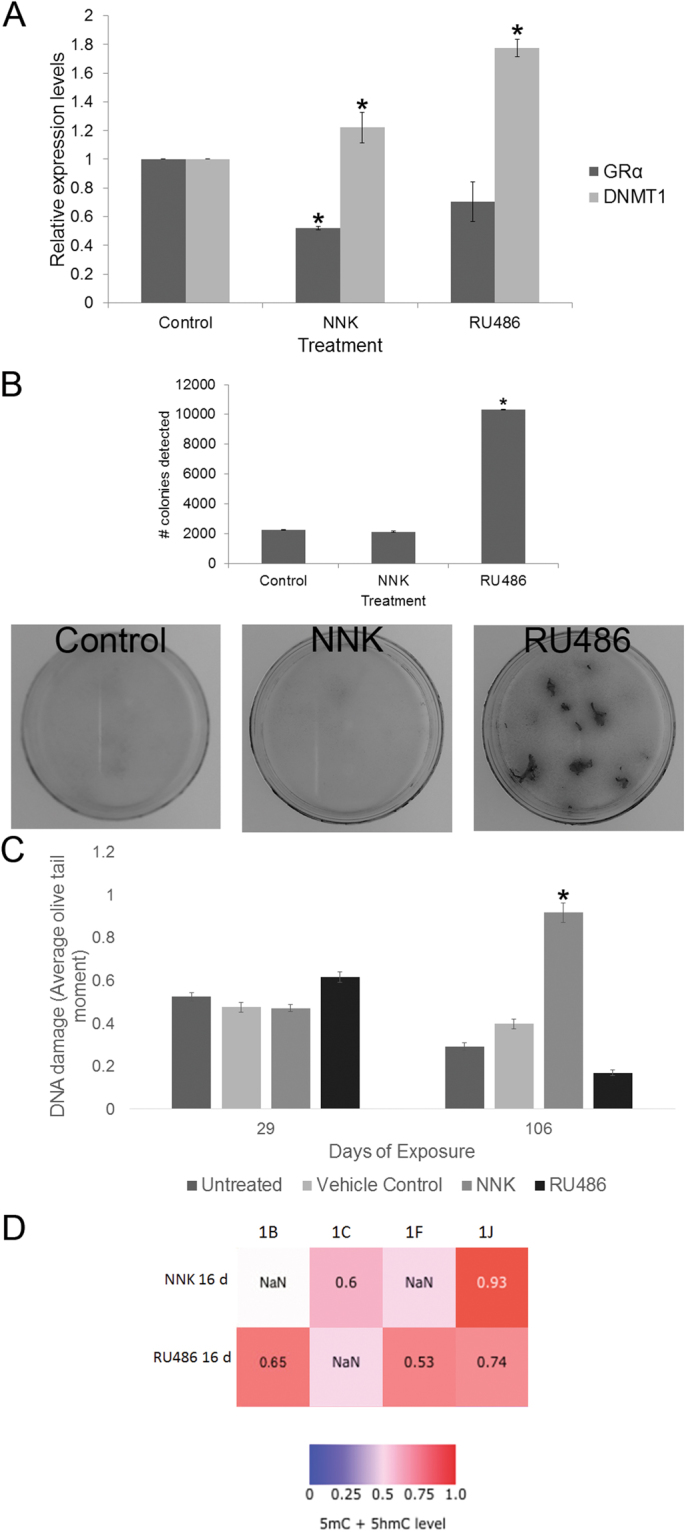
Long term exposure of MRC-5 cells to NNK results in a decrease in GRα and an increase in DNMT1 expression. Exposure to the GR antagonist, RU486, has the same effect. Clonogenic and comet analysis reveal that NNK has no effect on cell proliferation while RU486 enhances proliferation. By 106 days, NNK induces significant DNA damage. (**A**) Relative expression of GRα and DNMT1 in MRC-5 cells treated with either NNK or RU486 for 62 days (n = 3, p = 0.001 compared to the control). (**B**) Clonogenic assay performed on cells chronically treated with either NNK or RU486 for 86 days. (**C**) Comet assay showing extent of DNA damage after 29 and 106 days of chronic NNK or RU486 treatment (n = 300, p < 0.05 compared to other treatments). (**D**) Heat map of mean GR promoter methylation levels in MRC-5 cells treated with NNK or RU486 for 16 days. NaN – Not a Number i.e. the value is a mathematically undefined number.

We previously showed that restoring GR expression to GR deficient SCLC cells and xenografts results in apoptotic death, suggesting that loss of GR expression by methylation plays an important role in carcinogenesis^4,10,18^. To explore the role of GR and to compare against NNK exposure, we performed a long term exposure study in non-transformed lung fibroblast cells to either NNK or RU486. Longer term treatment (62 days) with NNK resulted in a significant increase in DNMT1 expression that was accompanied by a general decrease in GRα expression (Fig. 3A). The effects of NNK on DNMT1 expression were mirrored by RU486, a GR antagonist (Fig. 3A). Both NNK and RU486 reduced GRα expression although the RU486 effect did not reach significance. NNK exposure had little effect on cell proliferation after 86 days, while RU486 significantly stimulated cellular proliferation (Fig. 3B), implying alleviation of a repressive effect. DNA damage analysis using the comet assay showed that as expected, NNK treatment caused significant DNA damage after 106 days of exposure while RU486 appeared to have a protective effect (Fig. 3C). Although cells proliferated faster with RU486 treatment, cells exposed to all treatments senesced after 42–46 doublings reinforcing the view that either NNK or RU486 treatment alone was insufficient for transformation. Interestingly, both NNK and RU486 treatment increased methylation at the GR promoters after 16 days compared to the control (Fig. 3D).

## Discussion

Exposure to the cigarette carcinogen NNK is known to lead to the development of lung cancer, partly through the hypermethylation of numerous TSG promoters via over-expression of DNMT1^20^. DNMT1 has been shown to be specifically over-expressed in NSCLC lung tumours compared to normal cells where DNMT1 over-expression resulted in increased methylation of the TSGs, *FHIT, p*1*6*^*INK4a*^ and *RARβ*^21^. This also correlated with smoking status and poor prognosis of lung cancer patients^15^.

Our data reveal that DNMT1 expression is significantly higher in the representative SCLC than NSCLC cell line. Furthermore, as shown previously for NSCLC cell lines^15^, NNK treatment further increased DNMT1 expression in our SCLC cell line, DMS79. While DNMT1 is expressed at high levels in both types of lung cancer the differing levels of expression may play a role in generating the distinct methylation profiles recently identified through a genome wide methylation analysis study^22^. Importantly, DNMT1 was recruited to the GR promoter 1 F and 1B, in line with our previous work showing that re-expression of the GR in SCLC cells mediated by treatment with a demethylating agent is primarily driven by demethylation of promoter 1 F^11^.

SCLC and NSCLC tumours originate from cells of the bronchial epithelium^23^, yet it is not known whether SCLC and NSCLC originate from similar or different cell types. The specific cell type/s that give rise to SCLC have not been conclusively shown, however there is extensive genetic damage in the normal bronchial epithelium surrounding SCLC tumours^24^, with recent evidence supporting that the development of solid tumours is driven by concurrent mutations in surrounding stromal cells^25^. Fibroblasts are the most abundant stromal cell type, and in NSCLCs, Vizoso *et al*.^25^ discovered a pattern of widespread DNA hypomethylation with region-specific hypermethylation within tumour-supporting fibroblasts that was in contrast to fibroblasts from unaffected tissue, yet similar to cells of the tumour^25^. At the earliest stages of carcinogenesis, even normal tissue may be subjected to epigenetic alterations to tumour progenitor genes such as the GR^26^. We thus considered this cell type as a good model for understanding NNK-induced effects on the GR in the lung. Since our previous work has shown that silencing of the GR plays an important role in tumourigenesis^4,18^, and since the GR has recently been identified as a novel TSG^12^, we analysed the effects of NNK on the untransformed lung fibroblast cell line, MRC-5. Short term NNK treatment significantly reduced GR mRNA and protein levels due to accumulation of DNMT1 at GR promoters 1B and 1F. This mimics the pattern seen in the SCLC cell line and reveals a direct link between smoking and methylation of the GR in lung cancer. Analysis of the NGS data revealed heavy methylation of all the promoter regions, reinforcing that the GR is a target for NNK-induced methylation. We have previously shown that in MRC-5 cells, promoter 1*C* shows the highest endogenous expression, followed by promoters 1B, then 1F and 1J^11^. Furthermore, the ubiquitously expressed 1B and 1C promoters^27^ are the most active in GR transcription^6,28^. Our data therefore suggest that promoter 1B and 1C, possibly because they are most important to silence *GR* expression, are the major targets for NNK-induced methylation. Conversely, the extensive methylation observed at promoter 1J may have little effect on *GR* expression since MRC-5 cells show minimal usage of this promoter^11^. Furthermore, Lin *et al*.^15^ reported that NNK-induced DNMT1 protein accumulation was restored to basal levels in as little as 2 h post NNK-withdrawal, suggesting that depletion of DNMT1 was hindered or, that methylation may remain even after DNMT1 returns to normal.

Since the GR appears to be a target for NNK-induced methylation and thus gene silencing, we attempted to determine whether long-term treatment of MRC-5 cells with NNK would have a similar effect to treatment with a GR antagonist, RU486. This potent GR antagonist can also confer partial agonist activity to the receptor^29,30^. However RU486 cannot activate fully either GR-mediated chromatin remodelling or gene transcription^31,32^. Thus, RU486 was used in preference to GR knock-down with siRNA (as complete loss of the GR can affect mitosis through a ligand-independent mechanism^12^) to modulate GR activity. Chronic NNK or RU486 exposure for 62 days had similar effects on elevating DNMT1 expression. However, treatment with NNK showed little effect on cellular proliferation, whereas RU486 stimulated proliferation. The effects of RU486 exposure appear to be cell type specific. RU486 treatment inhibits proliferation in endometrial cells^33^ and hepatic cells^34^ but is neuroprotective in Purkinje cells, protecting against apoptosis^35^. Further, it was suggested that the ability of RU486 to inhibit apoptosis in hepatic cells may enhance the potential for hepatocellular carcinoma^34^. After 29 days of exposure, the RU486 treated cells showed no signs of DNA damage. By 106 days of exposure NNK-treated cells, as expected, showed high levels of DNA damage. However, RU486 treated cells appeared to be protected against DNA damage. Since we have previously shown that re-expression of the GR leads to apoptosis, it may be that chronic antagonism of the GR acts to prevent apoptosis. Similar to the effects on gene expression, chronic exposure to both NNK and RU486 indeed affected methylation of the GR promoters.

Taken together our data identify the recently described lung cancer TSG gene, the GR, as a target for NNK-induced methylation, and silencing, thereby providing a direct mechanistic link between tobacco smoke exposure, and reduced GR expression.

## Methods

### Cell culture and treatments

A549 (NSCLC), DMS79 (SCLC) and MRC-5 cells were cultured as described in Singh *et al*.^11^. All cell lines were sourced from the American Type Culture Collection. Where applicable, cells were treated short-term with 10 µmol/L NNK (Sigma) and/or vehicle (DMSO, Sigma) for 24, 48 or 72 h, or chronically with 10 µmol/L NNK and/or vehicle and 100 nmol/L RU486 (Sigma) for up to 106 days.

### Quantitative real-time PCR (qPCR) gene expression analysis

RNA was isolated from treated or untreated cells (RNeasy Mini Kit, Qiagen) and cDNA was synthesised (SensiFast cDNA Synthesis Kit, Bioline). qPCR was performed in a 7500 Real Time PCR System (Applied Biosystems) using SYBR Select Master Mix for CFX (Applied Biosystems) or 5x HOT FIREPol EvaGreen qPCR Mix Plus (no ROX) (Solis BioDyne), as per the manufacturers’ instructions. Primer sequences are as follows: DNMT1 F 5′GAGGAAGCTGCTAAGGACTAGTTC3′, R 5′ACTCCACAATTTGATCACTAAATC3′; GRα F 5′CCATTGTCAAGAGGGAAGGA3′, R 5′CAGCTAACATCTCGGGGAAT3′; GR promoters 1B F 5′GCCGGCACGCGACTCC3′; 1 C F 5′GCTCCTCTGCCAGAGTTGAT3′; 1E F 5′CGTGCAACTTCCTTCGAGT3′; 1F F 5′GTAGCGAGAAAAGAAACTGG3′; 1J F 5′CCGGGGTGGAAGAAGAG3′; Exon 2 R 5′CAGTGGATGCTGAACTCTTGG3′; GAPDH F 5′GAAGGTGAAGGTCGGAGT3′, R 5′GAAGATGGTGATGGGATTTC3′; β-actin F 5′GGCCACGGCTGCTTC3′, R 5′GTTGGCGTACAGGTCTTTGC3′. qPCR data were analysed using the 2^−∆∆CT^ method^36^ relative to the geometric mean of the reference genes GAPDH and β-actin^37^.

### Chromatin immunoprecipitation (ChIP)

Cells were harvested at a final density of 2 × 10^7^ and subjected to the ChIP assay (EZ-ChIP™, Upstate, Millipore) as per the manufacturer’s instructions. Monoclonal mouse anti-DNMT1 (Imgenex) at a concentration of 2 μg per reaction was used for immunoprecipitation. The SensiFast SYBR No-Rox Kit (Bioline) was used for qPCR along with 2 µL of the ChIP-purified DNA as per the manufacturer’s instructions and cycling conditions. Primers are as follows: 1B F 5′GGGCCCAAAGTACGTATGC3′, R 5′CGAGTTGCGTGAAGTGTGTC3′; 1F F 5′CCCCTTTCGAAGTGACACAC3′, R 5′CCACCGAGTTTCTCCAGTTT3′; 1J F 5′TATGAACGTGATAGGGTGAGCA3′, R 5′CAAGTTGCAGGCGAAATAGTAA3′; GAPDH F 5′TACTAGCGGTTTTACGGGCG3′, R 5′TCGAACAGGAGGAGCAGAGAGCGA3′. The relative GR promoter occupancy was determined using the 2^−∆∆CT^ method relative to GAPDH.

### Western blots

MRC-5 cells were lysed with RIPA buffer (Sigma) containing proteinase inhibitor cocktail and quantified using the Pierce™ BCA Protein Assay Kit (ThermoFisher Scientific). Proteins were separated on a 13.5% SDS-PAGE gel, blotted and probed with GR antibody (611226, BD Biosciences). The membranes were visualised post incubation with Bio-Rad Immun-Star™ HRP Chemiluminescent Kit. Total protein content was determined by staining the GR protein nitrocellulose membrane with 1x Ponceau S Staining solution for 5 min followed by a 30 s wash in destaining solution. Densitometric analyses of the GR protein band intensity was calculated relative to GAPDH protein.

### Clonogenic assay

Cells (1000) from day 74 of the chronic experiment were harvested and cultured in cell culture dishes for 12 days to achieve 6 cell divisions. The cells were then fixed with cold methanol (Merck) and stained with 0.5% crystal violet (Merck). Images of the dishes were captured and the numbers of spots/colonies were determined using Icy - Spot Detector^38^.

### Comet assay

MRC-5 cells were harvested from days 29 and 106 of the chronic treatment. Cells were prepared for comet assay analyses as per Weber *et al*.^39^. Comet slides were visualised using a Nikon Eclipse E400 fluorescent microscope with an excitation filter of 510–560 nm and a barrier filter of 590 nm. Images were captured at 200x magnification using a Nikon Digital Sight DS-Fi2 digital camera and Nikon NIS Elements D Software version 4.20. Comet images were analysed for DNA damage, determined by measuring the Olive Tail Moments, using Comet Assay Software Project (CASP) version 1.2.2.

### Next-generation sequencing (NGS)

DNA was extracted from MRC-5 cells (DNeasy® Blood & Tissue Kit, QIAGEN, UK) and bisulfite-converted (EZ DNA Methylation™ Kit, Zymo Research). Bisulfite-specific PCR was performed in a Veriti 96 Well Thermal Cycler (Applied Biosystems) using Zymo*Taq*™ DNA polymerase (Zymo Research), as per the manufacturer’s instructions. Primer sequences are as follows: 1B F 5′*TGACTGGAGTTCAGACGTGTGCTCT TCCGATCT*GGGAGGGGTGGGGGTTGAATTTGGTAGG3′, R 5′*ACACTCTTTCCCCACACGACGCTCTTCCGATCT*C CCTCTAAAAAAACTTCAAAAAAAAAAC3′; 1C F 5′*TGACTGGAGTTC AGACGTGTGCTCTTCCGATCT*GGGGGTG GAGTGGGAG3′, R 5′*ACACTCTTTCCCCACACGACGCTCTTCCGATCT*CTTCT TACCTCTAACAAAAAAACC3′; 1F F 5′*TGACTGGAGTTCAGACGTGTGCTCTTCCGATCT*GTTTTTTTTTTGAAGT TTTTTTAGAGGG3′, R 5′*ACACTCTTTC CCCACACGACGCTCTTCCGATCT*CC CCCAACTCCCCAAAAAAAAAAA TAAC3′; 1J F 5′*TGACTGGAGTTCAGACG TGTGCTCTTCCGATCT*GGATTGAGGGGGAAGTTTTTAATAGG3′, R 5′*ACACTCTTTCCCCACACGACGCTCTTCCG ATCT*CTAAATTTCTTTACACTTTTTTTTTATTAT3′; IGF2 DMR F 5′TAATTTATTTAGGGTGGTGTT3′, R 5′TCCAAA CACCCCCACCTTAA3′. Primers were designed with adaptor sequences (indicated in italics) at the 5′ end to achieve the amplicon size of ~300 bp required for sequencing. The IGF2 DMR primer set was not modified as this reaction served only as a positive control thus was not sequenced. Bisulfite-converted DNA was purified using the QIAquick® Gel Extraction Kit (QIAGEN, UK). The DNA samples were sent to Inqaba Biotec for sequencing using the MiSeq v3 platform (Illumina®).

Quality control on the data was performed using Galaxy software (version 15.10.dev). BiQ Analyzer HiMod (Becker *et al*.^40^) was then used to determine the methylation levels of the amplified promoters in each treatment relative to the 72 h vehicle-treated cells.

### Statistical analyses

Data from qPCR, ChIP and Western blot experiments were analysed by one-way analysis of variance (ANOVA). Comet assay data were analysed by two-way ANOVA. Post hoc Tukey tests were run for multiple comparisons. All statistical analyses were performed using IBM SPSS Statistics version 21.

## References

[1] Jackman DM, Johnson BE (2005). Small-cell lung cancer. Lancet.

[2] Stewart MF, Crosby SR, Gibson S, Twentyman PR, White A (1989). Small cell lung cancer cell lines secrete predominantly ACTH precursor peptides not ACTH. Br. J. Cancer.

[3] Ray DW, Littlewood AC, Clark AJL, Davis JRE, White A (1994). Human small cell lung cancer cell lines expressing the proopiomelanocortin gene have aberrant glucocorticoid receptor function. J. Clin. Invest..

[4] Sommer P (2007). Glucocorticoid receptor overexpression exerts an antisurvival effect on human small cell lung cancer cells. Oncogene.

[5] Schmidt S (2004). Glucocorticoid-induced apoptosis and glucocorticoid resistance: molecular mechanisms and clinical relevance. Cell Death Differ..

[6] Turner JD, Muller CP (2005). Structure of the glucocorticoid receptor (NR3C1) gene 5′ untranslated region: identification, and tissue distribution of multiple new human exon 1. J. Mol. Endocrinol..

[7] Zhou J, Cidlowski JA (2005). The human glucocorticoid receptor: one gene, multiple proteins and diverse responses. Steroids.

[8] Turner JD, Pelascini LPL, Macedo JA, Muller CP (2008). Highly individual methylation patterns of alternative glucocorticoid receptor promoters suggest individualized epigenetic regulatory mechanisms. Nucleic Acids Res..

[9] Turner JD (2010). Transcriptional control of the glucocorticoid receptor: CpG islands, epigenetics and more. Biochem. Pharmacol..

[10] Kay P (2011). Loss of glucocorticoid receptor expression by DNA methylation prevents glucocorticoid induced apoptosis in human small cell lung cancer cells. PLoS One.

[11] Singh N (2014). The N-terminal transactivation domain of the glucocorticoid receptor mediates apoptosis of human small cell lung cancer cells. Genes Chromosomes Cancer.

[12] Matthews LC (2015). Glucocorticoid receptor regulates accurate chromosome segregation and is associated with malignancy. Proc. Natl. Acad. Sci. USA.

[13] Jones PA, Baylin SB (2002). The fundamental role of epigenetic events in cancer. Genetics.

[14] Esteller M (2007). Epigenetic gene silencing in cancer: the DNA hypermethylome. Hum. Mol. Gen..

[15] Lin RK (2010). The tobacco-specific carcinogen NNK induces DNA methyltransferase 1 accumulation and tumor suppressor gene hypermethylation in mice and lung cancer patients. J. Clin. Invest..

[16] Robertson KD (2001). DNA methylation, methyltransferases, and cancer. Oncogene.

[17] Nephew KP, Huang TH (2003). Epigenetic gene silencing in cancer initiation and progression. Cancer Lett..

[18] Sommer P (2010). Glucocorticoid receptor over-expression promotes human small cell lung cancer apoptosis *in vivo* and thereby slows tumour growth. Endocr. Relat. Cancer.

[19] Jacobs JP, Jones CM, Baille JP (1970). Characteristics of a human diploid cell designated MRC-5. Nature.

[20] Vuillemenot BR, Hutt JA, Belinsky SA (2006). Gene promoter hypermethylation in mouse lung tumors. Mol. Cancer Res..

[21] Lin RK (2007). Alteration of DNA methyltransferases contributes to 5′CpG methylation and poor prognosis in lung cancer. Lung Cancer.

[22] Karlsson A (2014). Genome-wide DNA methylation analysis of lung carcinoma reveals one neuroendocrine and four adenocarcinoma epitypes associated with patient outcome. Clin. Cancer Res..

[23] Hopkins-Donaldson S (2003). Silencing of death receptor and caspase-8 expression in small cell lung carcinoma cell lines and tumors by DNA methylation. Cell Death Differ..

[24] Wistuba II (2000). Molecular changes in the bronchial epithelium of patients with small cell lung cancer. Clin. Cancer Res..

[25] Vizoso M (2015). Aberrant DNA methylation in non-small cell lung cancer-associated fibroblasts. Carcinogenesis.

[26] Shi L (2017). Regulatory roles of epigenetic modulators, modifiers and mediators in lung cancer. Semin. Cancer Biol..

[27] Nunez BS, Vedeckis WV (2002). Characterization of promoter 1B in the human glucocorticoid receptor gene. Mol. Cell. Endocrinol..

[28] Alt SR (2010). Differential expression of glucocorticoid receptor transcripts in major depressive disorder is not epigenetically programmed. Psychoneuroendocrinology.

[29] Jackson TA (1997). The partial agonist activity of antagonist-occupied steroid receptors is controlled by a novel hinge domain-binding coactivator L7/SPA and the corepressors N-CoR or SMRT. Mol. Endocrinol..

[30] Smith CL, Nawaz Z, O’Malley BW (1997). Coactivator and corepressor regulation of the agonist/antagonist activity of the mixed antiestrogen, 4-hydroxytamoxifen. Mol. Endocrinol..

[31] Skafar DF (1991). Differences in the binding mechanism of RU486 and progesterone to the progesterone receptors. Biochemistry.

[32] Peeters BWMM (2008). Differential effects of the new glucocorticoid receptor antagonist ORG 34517 and RU486 (mifepristone) on glucocorticoid receptor nuclear translocation in the AtT20 cell line. Ann. N Y Acad. Sci..

[33] Murphy AA (2000). RU486-induced growth inhibition of human endometrial cells. Fertil. Steril..

[34] Youssef JA, Badr MZ (2003). Hepatocarcinogenic potential of the glucocorticoid antagonist RU486 in B6C3F1 mice: effect on apoptosis, expression of oncogenes and the tumor suppressor gene p53. Mol. Cancer.

[35] Ghoumari AM (2003). Mifepristone (RU486) protects Purkinje cells from cell death in organotypic slice cultures of postnatal rat and mouse cerebellum. Proc. Natl. Acad. Sci. USA.

[36] Livak KJ, Schmittgen TD (2001). Analysis of relative gene expression data using real-time quantitative PCR and the 2^-ΔΔCT^ method. Methods.

[37] Vandesompele J (2002). Accurate normalization of real-time quantitative RT-PCR data by geometric averaging of multiple internal control genes. Genome Biol..

[38] de Chaumont F (2012). Icy: an open bioimage informatics platform for extended reproducible research. Nature Methods.

[39] Weber S (2013). Comet assay and air-liquid interface exposure system: A new combination to evaluate genotoxic effects of cigarette whole smoke in human lung cell lines. Toxicol. In Vitro.

[40] Becker, D. *et al*. BiQ Analyzer HiMod: an interactive software tool for high-throughput locus-specific analysis of 5-methylcytosine and its oxidized derivatives. *Nucleic Acids Res.* **42(Web server issue)**, W501–W507, 10.1093/nar/gku457 (2014). 10.1093/nar/gku457 PMC4086109 24875479

